# No additive genetic variance for tolerance to ethynylestradiol exposure in natural populations of brown trout (*Salmo trutta*)

**DOI:** 10.1111/eva.12767

**Published:** 2019-01-28

**Authors:** Lucas Marques da Cunha, Anshu Uppal, Emily Seddon, David Nusbaumer, Etienne L.M. Vermeirssen, Claus Wedekind

**Affiliations:** ^1^ Department of Ecology and Evolution, Biophore University of Lausanne Lausanne Switzerland; ^2^ Swiss Centre for Applied Ecotoxicology Eawag‐EPFL Dübendorf Switzerland

**Keywords:** additive genetic variance, EE2, Estrogen, rapid evolution, Salmonidae

## Abstract

One of the most common and potent pollutants of freshwater habitats is 17‐alpha‐ethynylestradiol (EE2), a synthetic component of oral contraceptives that is not completely eliminated during sewage treatment and that threatens natural populations of fish. Previous studies found additive genetic variance for the tolerance against EE2 in different salmonid fishes and concluded that rapid evolution to this type of pollution seems possible. However, these previous studies were done with fishes that are lake‐dwelling and hence typically less exposed to EE2 than river‐dwelling species. Here, we test whether there is additive genetic variance for the tolerance against EE2 also in river‐dwelling salmonid populations that have been exposed to various concentrations of EE2 over the last decades. We sampled 287 adult brown trout (*Salmo trutta*) from seven populations that show much genetic diversity within populations, are genetically differentiated, and that vary in their exposure to sewage‐treated effluent. In order to estimate their potential to evolve tolerance to EE2, we collected their gametes to produce 730 experimental families in blockwise full‐factorial in vitro fertilizations. We then raised 7,302 embryos singly in 2‐ml containers each and either exposed them to 1 ng/L EE2 (an ecologically relevant concentration, i.e., 2 pg per embryo added in a single spike to the water) or sham‐treated them. Exposure to EE2 increased embryo mortality, delayed hatching time, and decreased hatchling length. We found no population differences and no additive genetic variance for tolerance to EE2. We conclude that EE2 has detrimental effects that may adversely affect population even at a very low concentration, but that our study populations lack the potential for rapid genetic adaptation to this type of pollution. One possible explanation for the latter is that continuous selection over the last decades has depleted genetic variance for tolerance to this synthetic stressor.

## 1 INTRODUCTION

The resilience of natural populations depends on whether they can show rapid evolutionary responses to novel selection pressures (Hendry, Gotanda, & Svensson, 2017). For a rapid evolutionary response to occur, populations must harbor heritable genetic variance for tolerance to the selection pressure, because adaptation by favorable new mutations is usually a slow process (Barrett & Schluter, 2008). A typical example of novel selection pressures is pollution by endocrine‐disrupting chemicals (Corcoran, Winter, & Tyler, 2010; Johnson & Sumpter, 2014), and among them pollution by the synthetic 17α‐ethynylestradiol (EE2) (Corcoran et al., 2010; Johnson & Sumpter, 2014). This steroid is a compound of oral contraceptives and reaches the environment through household sewage (Chèvre, 2014; Ternes, Kreckel, & Mueller, 1999). During the sewage treatment process, EE2 removal is expected to be on average 68% (Johnson et al., 2013). Thus, rivers that carry treated sewage effluent typically also carry EE2. Modeling work suggests that EE2 is present at 10 pg/L or higher in 20% of the entire European river network (Johnson et al., 2013). Concentrations around 1 ng/L have been measured in surface waters worldwide (data summarized in table 4 of Tiedeken, Tahar, McHugh, & Rowan, 2017).

In fish, exposure to EE2 can affect growth, survival, and even sex differentiation (Devlin & Nagahama, 2002); that is, EE2 can disturb gonad development (Caldwell, Mastrocco, Anderson, Länge, & Sumpter, 2012; Leet, Gall, & Sepúlveda, 2011) with potential consequences for individual and population fitness (Cotton & Wedekind, 2007; Wedekind, 2017). Embryos and larvae seem to be most susceptible to the toxicity of EE2 (Aris, Shamsuddin, & Praveena, 2014), especially so in salmonids. For example, in embryos and larvae of the Atlantic salmon (*Salmo salar*), a 4‐day exposure to low concentrations of EE2 led to increased vitellogenin expression (an indicator of the biological effect of estrogenic compounds; Duffy, Iwanowicz, & McCormick, 2014). Moreover, Brazzola, Chèvre, and Wedekind (2014) exposed embryos of two species of whitefish (*Coregonus palaea* and *C. albellus*) to low concentrations of EE2 (from 1 to 100 ng/L) and found EE2 at all concentrations to delay hatching and to reduce embryo survival and growth. Luca et al. (in preparation) confirmed the toxicity of EE2 in whitefish and linked it to changes in the metabolic rates during embryogenesis, and Selmoni et al. (2017) found EE2 to delay gonad development in grayling larvae (*Thymallus thymallus*).

Salmonids are external fertilizers with no parental care. This allows for full‐factorial in vitro fertilizations and the rearing of experimental crosses under various experimental conditions (e.g., Jacob et al., 2007). The sire effect in such rearing experiments is then a useful estimator of the additive genetic effect of the embryos’ reaction to a stressor (Lynch & Walsh, 1998). This makes salmonids a powerful model for quantitative genetic studies. Brazzola et al. (2014) and Luca et al. (in preparation) used such full‐factorial in vitro breeding experiments to estimate the variance components of tolerance to EE2. They found additive genetic variance for tolerance in three independent analyses on two whitefish populations; that is, they found that the susceptibility to EE2 depended on genetic characteristics of individuals that are directly heritable. However, Brazzola et al. (2014) and Luca et al. (in preparation) studied lake‐spawning salmonids that may typically be less exposed to EE2 than river‐spawning fish, as observed for other micropollutants (e.g., Moschet, Götz, Longrée, Hollender, & Singer, 2013). The stronger the selection pressure, the more likely it is that genetic variation on loci linked to the genes under selection erodes over time (Hendry et al., 2011; Lynch & Walsh, 1998), and the more likely it is that adaptation has happened. It therefore remains to be tested whether river‐dwelling salmonid populations that have most likely been exposed to higher EE2 concentrations since the launch of oral contraceptives (i.e., since the 1960s) are as susceptible to EE2 as whitefish, and whether they also show additive genetic variance for tolerance to this pollutant.

**Figure 1:**
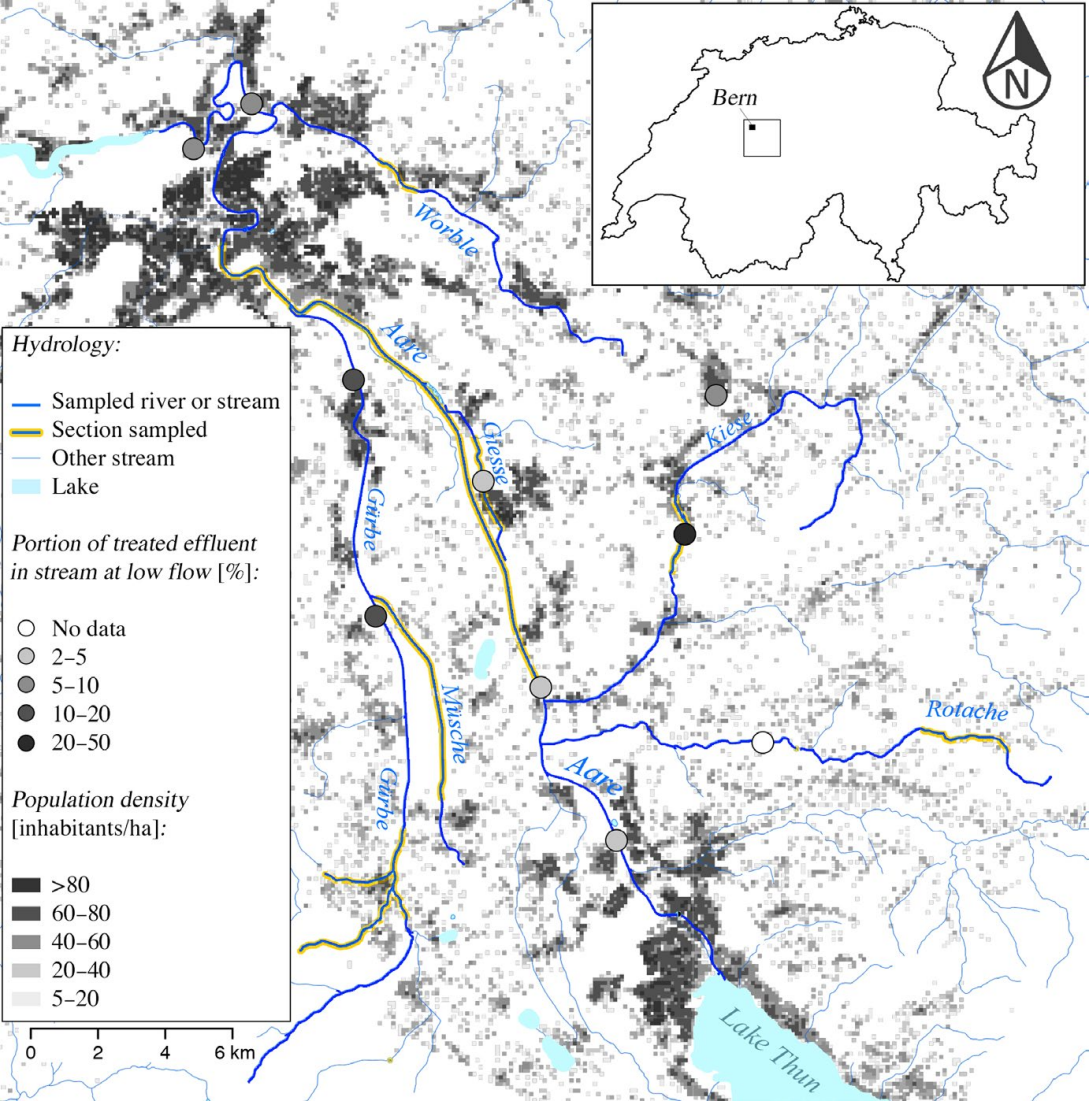
Simplified map of the study area (Aare river system between Lake Thun and the city of Bern, the Aare is the outlet of Lake Thun). Adults were sampled in Aare, Worble, Giesse, Gürbe, Kiese, Müsche, and Rotache (see Supporting Information Table S1 for sampling dates). Circles indicate the location of sewage treatment plants and their gray value the percentage of treated effluent in the river or stream at low flow (%). Shades of gray indicate the human population density (inhabitants/hectare). The box in the upper right inlet indicates the location in Switzerland. Adapted from a map produced by the Swiss Confederation in collaboration with the cantons (www.map.geo.admin.ch, downloaded on November 1, 2018)

The brown trout (*Salmo trutta* L.) of the Aare river network in Switzerland have been extensively monitored and experienced a decline of over 50% over the past three decades (Burkhardt‐Holm, 2007). This decline may have multiple causes, including estrogen pollution (Burkhardt‐Holm et al., 2008). A series of studies focused on a part of this river network, that is, a ca. 30‐km‐long valley with various tributaries that differ in their ecology, to test for potential genetic factors that may have contributed to the observed decline. These studies found much genetic variation on selectively neutral loci within samples taken at different locations (Stelkens, Jaffuel, Escher, & Wedekind, 2012) and a high level of genetic divergence between samples taken from the different tributaries (Stelkens, Jaffuel et al., 2012). Habitat quality during embryo development (Stelkens, Pompini, & Wedekind, 2012) varies in these tributaries, and local populations display significant differences in some fitness‐relevant traits (Pompini, Clark, & Wedekind, 2013) that could not be linked to potential indicators of inbreeding (Clark, Stelkens, & Wedekind, 2013; Stelkens, Pompini, & Wedekind, 2014). The ecology of the various tributaries within this region varies in many respects, including the stream slope and the density of fish populations (Stelkens, Jaffuel et al., 2012), the degree of urbanization (Figure 1), the presence of sewage treatment plants, and the discharge of their effluent relative to the discharge of the stream (Figure 1). All of these factors may be useful proxies of EE2 contamination in the environment (Johnson et al., 2013; Tiedeken et al., 2017).

Here, we sampled brown trout from seven different locations within this region, that is, from the main river and six different tributaries. Although migration between most of these seven locations is possible, 19 of all 21 possible pairwise *F*_ST_ calculated from polymorphic microsatellite markers were significant, and there were significant population differences in body shapes (Stelkens, Jaffuel et al., 2012). We have no historic EE2 measurements from the region, but from the information that we have about effluent loads and stream discharges (Figure 1), we conclude that these streams must have been differently exposed to EE2 throughout their recent history. The aims of the present study were to test (a) whether a low and ecologically relevant exposure to EE2 is toxic to embryos (as it is the case in whitefish; Brazzola et al., 2014), (b) whether populations differ in their susceptibilities to EE2, and (c) whether there is additive genetic variance for tolerance to EE2 within the study region.

## 2 MATERIAL AND METHODS

### 2.1 Experimental design and embryo rearing

Adult males (*N* = 142) and females (*N* = 145) were sampled during their spawning season from seven populations including the main river Aare and its tributaries Giesse, Gürbe, Kiese, Müsche, Rotache, and Worble (Figure 1; Supporting Information Table S1). These fish were transported to the *Fischereistützpunkt Reutigen* where they were held until the experimental crosses could be done. They were then returned to the sampling site and released.

The fish were stripped for their gametes that were used for in vitro blockwise full‐factorial fertilizations (Lynch & Walsh, 1998) within each population (Supporting Information Figure S1A). Twenty‐three of 29 breeding blocks were 5 × 5 blocks (i.e., five females crossed with five males in all possible combinations to produce 25 sibgroups). The remaining blocks included one 3 × 5, one 4 × 5, one 5 × 6 block, and three 6 × 5 blocks, depending on the availability of individuals (four males were unintentionally used twice in different blocks each). This resulted in 730 sibgroups. Freshly fertilized eggs were allowed to harden for 2 hr (Supporting Information Figure S1B) before samples of each sibgroup were transported to the laboratory. In the laboratory, 10 or 11 freshly fertilized eggs per sibgroup (*N*_total_ = 7,302) were used for the present study (further details on the experimental design can be found in Supporting Information Table S2) and the remaining eggs were used for another study (Marques da Cunha et al., in preparation). The eggs were washed as in von Siebenthal, Jacob, and Wedekind (2009) and singly distributed to polystyrene 24‐well plates (Greiner Bio‐One, Austria) filled with 1.8 ml of autoclaved standardized water per well (OECD, 1992). Plates were incubated in a climate chamber at 4.6°C. No water changes were performed throughout the experiment. This experimental protocol has been developed for salmonid embryos and has been repeatedly and successfully used in stress tolerance tests (e.g., Clark, Pompini, Marques da Cunha, & Wedekind, 2014; von Siebenthal et al., 2009; Wilkins, Marques da Cunha, Menin et al., 2017).

### 2.2 Treatment preparation, exposure, and trait measurements

A spike solution of 10 ng/L of analytical 17α‐ethynylestradiol (Sigma‐Aldrich, USA) was prepared through a 3‐step serial dilution. Because EE2 is poorly soluble in water, absolute ethanol (VWR International, USA) was used for the first step of the dilutions. This led to a concentration of 0.004% of ethanol in the final EE2 spike solution. Analogously, a control spike solution with the same concentration of ethanol but without EE2 was prepared. All of the dilutions were prepared with autoclaved standardized water (OECD, 1992). One day post fertilization, either 0.2 ml of the EE2 or 0.2 ml of the control spike solution was added to the wells for a final volume of 2 ml. The nominal concentrations in the wells were 1 ng/L of EE2 and 0.0004% of ethanol for EE2‐exposed embryos (i.e., a total content per well of 2 pg EE2) and 0.0004% of ethanol for sham‐treated individuals.

After fertilization success was assessed (15 days post fertilization), embryos were monitored daily and their mortality or hatching was noted. At the day of hatching, embryos were singly transferred to 12‐well plates (Greiner Bio‐One) filled with 3 ml of autoclaved standardized water (OECD, 1992); that is, there was no EE2 treatment at that stage (see Supporting Information Figure S1C for an example of a plate with larvae). These 12‐well plates were scanned for body measurements (Epson Perfection V37, Japan), that is, hatchling length and yolk sac length and width at hatching (see Supporting Information Figure S2 for an example of the measurements). After 24 days, the plates were again scanned for the same trait measurements. Larval growth was calculated as larval length at 24 days post hatching minus length at hatching. Yolk sac volume at hatching was calculated as in Jensen et al. (2008). All of the trait measurements were performed with ImageJ (http://rsb.info.nih.gov/ij/).

### 2.3 Statistical analyses and extraction of variance components

Embryo survival was analyzed as a binomial response variable in generalized linear mixed models (GLMM) fitted with maximum likelihood, and hatching time, hatchling length, yolk sac volume at hatching, and larval growth as continuous response variables in linear mixed models (LMM). LMMs were first fitted with maximum likelihood for testing the significance of the fixed effect treatment. The models were then refitted with restricted maximum likelihood for testing the significance of random effects with the package lmerTest (Kuznetsova, Brockhoff, & Christensen, 2017). Because embryos were raised singly, all the trait measurements were collected for each individual. Therefore, the replication unit in the statistical models is each embryo. We started with reference models that included treatment as a fixed effect and population, sire, and dam as random effects. The significance of the model terms was obtained by comparing a model including or lacking the term of interest to the reference model with likelihood ratio tests (LRT) and Akaike information criterion (AIC).

Variance components were extracted from mixed‐effect models and were used to calculate the components of phenotypic variation (Lynch & Walsh, 1998). Assuming that epistasis is negligible, additive genetic variance (*V*_A_) was calculated as four times the sire component of variation and dominance genetic variance (*V*_D_) as four times the sire × dam component. Narrow‐sense heritability estimates (*h* ^2^) were calculated as in Lynch and Walsh (1998) by dividing *V*_A _by the total phenotypic variance. The coefficients of additive genetic variation (CV_A_) were calculated as in Houle (1992), that is, dividing the square root of *V*_A_ by the mean of each trait and multiplying this with 100. Finally, the mean‐scaled additive genetic variance (*I*_A_) was calculated by dividing *V*_A_ by the square of the trait mean (Hansen, Pélabon, & Houle, 2011). All the statistical analyses were performed in R (R Development Core Team, 2015). Mixed‐effect models were performed with the lme4 package (Bates, Machler, Bolker, & Walker, 2015). The variance components were calculated and their significance tested with the *fullfact* package (Houde & Pitcher, 2016) based on maximum likelihood for the binary response variable embryo survival, and on restricted maximum likelihood for the continuous response variable. One of the 145 females used in the experiment showed exceptionally high and unexplained offspring mortality, that is, only 3 survivors out of 50 sampled embryos, and was therefore eliminated from all data analyses.

The experimental crosses were performed on 4 different days, once per week from mid‐November to mid‐December. Including date of breeding in the statistical models did not change any of the conclusions (results not shown).

### 2.4 Determining EE2 concentrations in 24‐well plates

We estimated embryo EE2 uptake and determined its persistence in the same model of polystyrene 24‐well plates as used in the main experiment. In total, 4,080 brown trout embryos (from other parents of the same populations as in the main experiment; seven 4 × 6 breeding blocks, i.e., 168 sibgroups of 28 females and 42 males) were raised in 170 24‐well plates (Greiner Bio‐One) with the same protocols as in the main experiment. Furthermore, 206 24‐well plates without embryos were prepared and analogously treated with EE2 or control spike solutions. Measuring EE2 in plates without embryos and comparing these measurements with plates that contained embryos allowed for estimations of embryo EE2 uptake. Water samples were collected at 5 time points across embryonic development and stored at −20°C for later EE2 measurements. The first time point was performed only in empty plates (18 plates per treatment) and was collected 30 min after the spike. For the following water samplings, the water of 21 entire plates was pooled per treatment (1,008 ml of water sample per treatment). These samplings were done in plates with and without embryos 7, 28, 56, and 84 days after exposures (hatching started a few days after the last sampling).

EE2 was quantified with liquid chromatography‐tandem mass spectrometry (LC‐MS/MS). First, the water samples were thawed and filtered with glass fiber filters. Then, sample volume and pH were set to 250 ml and 3, respectively. After that, 4 ng/L of 17α‐ethynylestradiol D4 was added to control for recovery and matrix effects. Water samples were enriched on LiChrolut EN/LiChrolut RP‐C18 cartridges (previously conditioned with hexane, acetone, methanol, and water at a pH of 3 as in Escher, Bramaz, Quayle, Rutishauser, and Vermeirssen (2008)). Cartridges were dried with nitrogen and eluted with acetone and methanol. Solvents were exchanged to hexane/acetone at a ratio of 65:35, and the extracts were passed through Chromabond Silica columns (Ternes, Stumpf et al., 1999). Finally, the volume of the extracts was set to 0.25 ml. LC‐MS/MS was performed with an Agilent 6495 Triple Quadrupole. The column used was an XBridge BEH C18 CP (2.5 µm, 2.1 mm × 75 mm). A gradient of acetonitrile/water was used for the liquid chromatography followed with a post‐column addition of ammonium fluoride solution. Mass transitions that were monitored are listed in Supporting Information Table S3. The LC‐MS/MS method also covered estrone (E1), 17β‐estradiol (E2), and bisphenol A (BPA). All three compounds were detected in the 24‐well plates with BPA at significant concentrations (Supporting Information Table S4).

## 3 RESULTS

### 3.1 EE2 water quantifications and uptake by embryos

**Figure 2:**
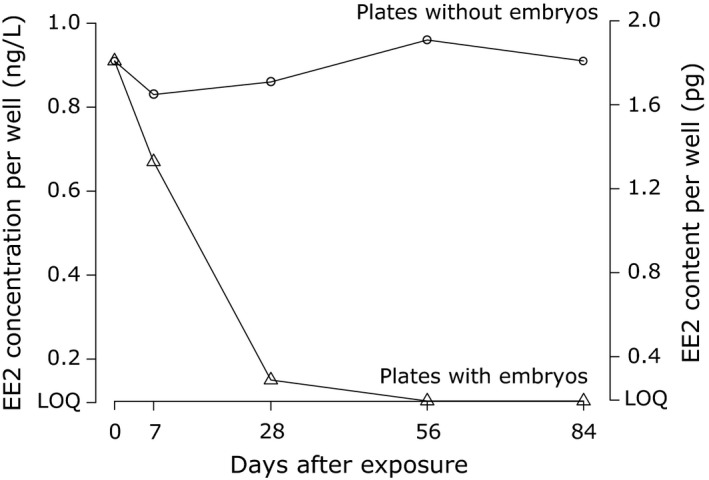
The persistence of 17α‐ethynylestradiol (EE2) in 24‐well plates with and without embryos across 5 time points. Triangles represent plates with embryos and circles without embryos. Sham‐treatment data points are not shown because they were always below LOQ (0.1 ng/L or a total well content of 0.2 pg). Symbols at LOQ level are present just for orientation; that is, they were below LOQ

The measured concentration of the EE2 and control spike solutions corresponded to the nominal concentrations. The EE2 spike solution concentration was 10.1 ng/L (nominal concentration = 10 ng/L), and the control spike solution concentration was lower than the limit of quantification (here LOQ = 0.05 ng/L). The concentrations of EE2 in water samples from 24‐well plates depended on the presence of embryos. In EE2‐spiked plates without embryos, the concentrations of EE2 remained near the expected nominal concentration (1 ng/L, i.e., a total content of 2 pg per well) throughout the observational period (Figure 2). In EE2‐spiked plates with embryos, the level of EE2 gradually declined from near the nominal concentration to the limit of quantification (Figure 2). Nearly all of this decline happened during the first month of embryogenesis (Figure 2). Control‐spiked plates with or without embryos did not show EE2 concentrations above LOQ (which was now at 0.1 ng/L EE2 for these measurements).

### 3.2 Population effects on tolerance to EE2

**Figure 3:**
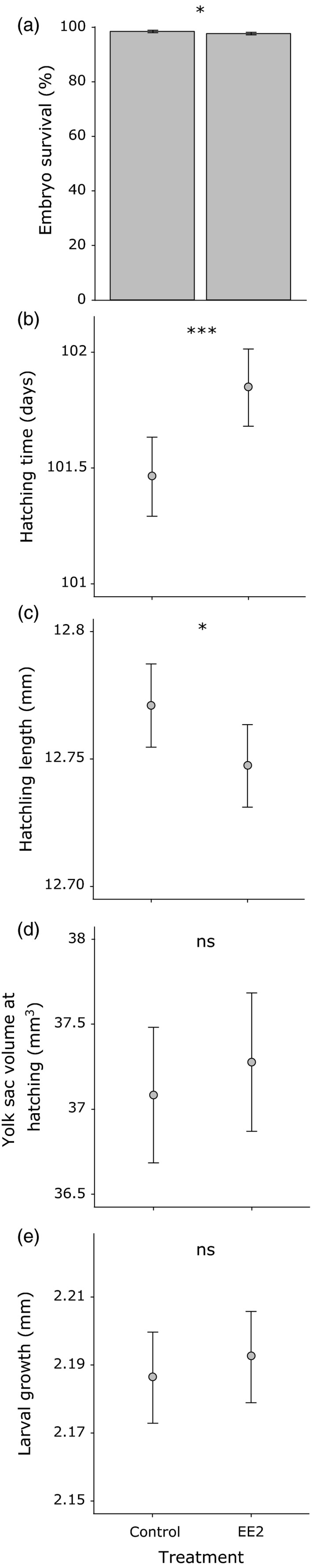
The effects of EE2 on embryo early phenotype: (A) embryo survival, (B) hatching time, (C) hatchling length, (D) yolk sac volume at hatching, and (E) larval growth. Bars (A) or circles (B–E) represent means of family means. Error bars are 95% confidence intervals. ***p < 0.001, *p < 0.05, and ns = p > 0.05. See Table 1 for statistics

Exposure to EE2 significantly reduced overall embryo survival (Table 1A) by 0.9 percent points (Figure 3a), led to an overall delay in hatching time of, on average, about half a day (Table 1B; Figure 3b) and reduced hatching length by 0.24% (Table 1C, Figure 3c). However, yolk sac volume at hatching and larval growth were not significantly affected by the EE2 treatment (Tables 1D–E; Figure 3d–e).

**Table 1:** The effects of treatment (exposure to EE2), population, sire, and dam on (A) embryo survival, (B) hatching time, (C) hatchling length, (D) yolk sac volume at hatching, and (E) larval growth

Model	Effect tested	AIC	*df*_LRT_	*X^2^*	*P*
*(A) Embryo survival*					
**t + sire + dam + pop**		968			
sire + dam + pop	t	970	1	4.5	**0.03**
t + sire + dam	pop	966	1	0.0	1
t + dam + pop	sire	971	1	5.2	**0.02**
t + sire + pop	dam	1,045	1	78.6	**<0.001**
t + sire + dam + t × pop	t × pop	972	2	0.0	1
t + sire + dam + pop + t × sire	t × sire	972	2	0.3	0.88
t + sire + dam + pop + t × dam	t × dam	967	2	5.3	0.07
*(B) Hatching time*					
**t + sire + dam + pop**		26,963			
sire + dam + pop	t	27,022	1	61.0	**<0.001**
t + sire + dam	pop	26,992	1	20.2	**<0.001**
t + dam + pop	sire	27,200	1	228.3	**<0.001**
t + sire + pop	dam	29,868	1	2,896.5	**<0.001**
t + sire + dam + t × pop	t × pop	26,973	2	0.1	0.98
t + sire + dam + pop + t × sire	t × sire	26,973	2	3.1	0.21
t + sire + dam + pop + t × dam	t × dam	26,973	2	4.1	0.13
*(C) Hatchling length*					
**t + sire + dam + pop**		2,869			
sire + dam + pop	t	2,876	1	8.8	**0.003**
t + sire + dam	pop	2,919	1	42.3	**<0.001**
t + dam + pop	sire	3,155	1	278.6	**<0.001**
t + sire + pop	dam	5,573	1	2,696.9	**<0.001**
t + sire + dam + t × pop	t × pop	2,879	2	0.8	0.66
t + sire + dam + pop + t × sire	t × sire	2,879	2	0.3	0.88
t + sire + dam + pop + t × dam	t × dam	2,879	2	0.1	0.93
*(D) Yolk sac volume at hatching*					
**t + sire + dam + pop**		31,069			
sire + dam + pop	t	31,069	1	2.0	0.16
t + sire + dam	pop	31,118	1	55.3	**<0.001**
t + dam + pop	sire	31,065	1	2.3	0.13
t + sire + pop	dam	35,615	1	4,552.1	**<0.001**
t + sire + dam + t × pop	t × pop	31,065	2	1.6	0.44
t + sire + dam + pop + t × sire	t × sire	31,065	2	−0.3	1
t + sire + dam + pop + t × dam	t × dam	31,065	2	0.4	0.82
*(E) Larval growth*					
**t + sire + dam + pop**		4,960			
sire + dam + pop	t	4,959	1	1.0	0.31
t + sire + dam	pop	4,987	1	18.3	**<0.001**
t + dam + pop	sire	5,022	1	52.8	**<0.001**
t + sire + pop	dam	5,249	1	280.0	**<0.001**
t + sire + dam + t × pop	t × pop	4,971	2	0.0	0.99
t + sire + dam + pop + t × sire	t × sire	4,971	2	0.2	0.88
t + sire + dam + pop + t × dam	t × dam	4,971	2	0.1	0.98

Note

Fixed effect: treatment (t); random effects: sire, dam, and population (pop). Likelihood ratio tests on mixed model regressions were used to compare a reference model (in bold) with models including or lacking the term of interest. Significant effects are highlighted in bold.

Populations significantly varied in most of the analyzed traits: Individuals from different populations displayed different hatching time, hatchling length, yolk sac volume at hatching, and larval absolute growth, but no significant effect was found for embryo survival (Table 1). The seven populations did not show specific tolerance to EE2 exposure for any of the analyzed early phenotypes; that is, the interaction “treatment × population” was never significant (Table 1).

### 3.3 Parental effects on tolerance to EE2

The parental effects (dam and sire identity) explained a large portion of the observed phenotypic variation. The dam effect was significant in all early traits, and the sire effect was significant for all traits except yolk sac volume at hatching (Table 1). Sires and dams did not display specific tolerance to EE2, that is, the interactions “treatment × sire” and “treatment × dam” were never significant (Table 1). Removing populations as a factor from the GLMM and LMM did not change these conclusions (results not shown). The variance components of phenotypic variation for each treatment, the narrow‐sense heritabilities, and the coefficients of variation are shown in Table 2.

**Table 2:** Maximum‐likelihood estimates of variance components for (A) embryo survival, and restricted maximum‐likelihood estimates of variance components for (B) hatching time, (C) hatchling length, (D) yolk sac volume at hatching, and (E) larval growth for each treatment, in addition to narrow‐sense heritabilities (*h* ^2^), mean‐scaled additive genetic variance (*I*_A_), and coefficients of additive genetic variation (CV_A_)

	*V*_A_	*V*_Dam_	*V*_D_	*V*_block_	*V*_res_	*h^2^*	*I*_A_	CV_A_
*(A) Embryo survival*								
Control	<0.01	1.0	156.6***	0	3.3	<0.01	<0.0001	<0.01
EE2	0	3.4	125.8***	0	3.3	0	0	0
*(B) Hatching time*								
Control	1.2***	2.2***	0	21.3***	3.7	0.04	0.0001	1.1
EE2	0.8***	2.3***	<0.01	20.6***	3.5	0.03	<0.0001	0.89
*(C) Hatchling length*								
Control	0.04***	0.07***	<0.01	0.09***	0.09	0.14	0.0002	1.49
EE2	0.03***	0.08***	0	0.09***	0.08	0.13	0.0002	1.44
*(D) Yolk sac volume at hatching*								
Control	0.03***	0.06***	0	0.07***	0.09	0.15	<0.0001	0.49
EE2	0.03***	0.06***	0	0.07***	0.08	0.15	<0.0001	0.47
*(E) Larval growth*								
Control	0.02***	0.01***	0	0.02***	0.1	0.10	0.0354	6.0
EE2	0.01	0.01***	0	0.02***	0.1	0.05	0.0017	4.1

Note

*V*_A_: additive genetic; *V*_Dam_: maternal; *V*_D_: dominance; *V*_res_: residual.

The significance of the variance components: ^*^ *p* < 0.05; ^**^ *p* < 0.01, ^***^ *p* < 0.001.

## 4 DISCUSSION

We studied the uptake of EE2 and its toxicity in brown trout from different populations, and we tested whether there are population differences in the responses and additive genetic variance for tolerance to this important pollutant of rivers and streams. We found that an ecologically relevant aqueous exposure at 1 ng/L was processed almost entirely by fish embryos within about a month, while EE2 dissolved in sterilized water was stable under our laboratory conditions for at least three months. We therefore conclude that EE2 was continuously taken up even at declining concentrations. Steroid hormones, such as EE2, can indeed be taken up by fish eggs and embryos (Bjerregaard, Lindholst, Korsgaard, & Bjerregaard, 2008), and steroids inside fish eggs and embryos are readily metabolized (Yeoh, Schreck, Feist, & Fitzpatrick, 1996). Our observation confirms that EE2 can be bioavailable at concentrations of 1 ng/L or lower as it has been previously shown (for reviews see Aris et al., 2014; Caldwell et al., 2012; Leet et al., 2011).

The aqueous exposure of only 1 ng/L EE2 turned out to be toxic to brown trout embryos. The effects we found are small and would usually be missed in studies based on smaller sample sizes or in experimental conditions that are less strict than ours. However, the observed reduced embryo survival, delayed hatching time, and decreased hatchling length can still affect population growth. Time and size of hatching are relevant to fitness in territorial salmonids such as brown trout, because larvae that emerge earlier from the gravel bed are more likely to establish a feeding territory and to outcompete their counterparts that emerge later (Skoglund, Einum, Forseth, & Barlaup, 2012). Moreover, larvae that emerge larger from the gravel bed have better swimming capacity and may, for example, be better at hunting or evading predators (Einum & Fleming, 2000).

Our estimates of the toxicity of EE2 may even be conservative. In our experimental setup, embryos were singly raised under conditions that are arguably close to optimal for their development (e.g., minimizing pathogen growth and mechanical stress). In the wild, embryos are typically exposed to a combination of various types of stressors, such as opportunistic microbes (Wilkins, Rogivue, Schütz, Fumagalli, & Wedekind, 2015) or other micropollutants (Chèvre, 2014; Moschet et al., 2014). If the toxicity of EE2 is amplified by further stress factors (e.g., Segner, Schmitt‐Jansen, & Sabater, 2014; Segner, Wahli, & Burkhardt‐Holm, 2012), the results from our experimental exposure underestimate the potential ecotoxicological relevance of EE2. Therefore, even low and ecologically relevant concentrations of EE2 are expected to induce selection.

Selection is expected to induce an evolutionary response if populations display additive genetic variance for tolerance to the stressor (Hendry et al., 2011). Our full‐factorial breeding experiment allowed us to estimate, via the sire effects, the overall additive genetic effects for several important life‐history traits. We found significant heritability for survival, hatching time, hatchling length, and growth; that is, some males were genetically superior to others. These findings support previous quantitative genetic studies on salmonids (Carlson & Seamons, 2008; Pitcher & Neff, 2007; Wedekind, Müller, & Spicher, 2001; Wilkins, Marques da Cunha, Glauser, Vallat, & Wedekind, 2017), including on the same brown trout populations we studied here (Clark et al., 2013; Pompini et al., 2013). However, it is the interaction term between the effect of a stressor and the sire effect that reveals additive genetic variance for tolerance to the stressor. These interaction terms were not significant for any of our potential fitness measures; that is, we found no significant additive genetic variance for tolerance to EE2.

An observed lack of additive genetic variance could potentially be a type II error (false negative). If so, our non‐significant findings would be explained by a lack of statistical power due to low sample size, low effect sizes (i.e., low toxicity of EE2), or large measurement error. We argue that none of these arguments are valid here: (a) Our experiment was conducted with a very large sample size of breeders (*N*_sires_ = 142, *N*_dams_ = 145) and a very large sample size of singly raised offspring (*N* = 7,302). Luca et al. (in preparation) and especially Brazzola et al. (2014) used much smaller sample sizes and still found significant additive genetic variance for tolerance to 1 ng/L EE2 in three independent tests on whitefish. (b) Our treatment produced significant EE2 effects on multiple life‐history traits. (c) Singly raised salmonid embryos are generally sensitive indicators of environmental stress. They have repeatedly been used in protocols that are comparable to ours to quantify the effects of, for example, various pathogens (Clark et al., 2014; von Siebenthal et al., 2009), organic pollution (Jacob, Evanno, von Siebenthal, Grossen, & Wedekind, 2010; Wedekind, Gessner, Vazquez, Maerki, & Steiner, 2010), various types of micropollutants (e.g., Brazzola et al., 2014; Clark, Pompini, Uppal, & Wedekind, 2016), or even waterborne cues emitted from infected eggs (Pompini et al., 2013; Wedekind, 2002).

Since a type II error is unlikely in our case, we conclude that the study populations lack additive genetic variation for tolerance to EE2. There are two possible explanations for such a result. First, the study populations may have never displayed additive genetic variance for tolerance to EE2. However, this kind of genetic variation is present in other salmonid species (Brazzola et al., 2014, Luca et al. in preparation), and our study populations display large effective population sizes (Stelkens, Jaffuel et al., 2012) and no significant signs of inbreeding depression (Stelkens, Pompini et al., 2012), and they show additive genetic variance for tolerance to other stressors (Pompini et al., 2013). The second possible explanation is that there was at one time significant genetic variance for tolerance to EE2 that has since been lost, for example, as a result of continuous selection over the last decades since the introduction of oral contraceptive pills and the use of EE2 in hormone replacement therapies.

A lack of genetic variance for tolerance to a stressor can be consequence of an erosion of genetic variance at loci linked to tolerance to the specific stressor. Such erosion could be produced by continuous selection (Hendry et al., 2011; Lynch & Walsh, 1998). Experimental evolution studies on stress tolerance have shown that a gradual increase in tolerance, and a gradual decrease in genetic diversity, can arise from continuous, multigenerational selection (e.g., Athrey, Leberg, & Klerks, 2007; Breckels, Garner, & Neff, 2014; Nowak et al., 2009). A major difference between the brown trout populations we studied here and the whitefish populations that Brazzola et al. (2014) and Luca et al. (in preparation) studied may be that whitefish are lake‐spawning and lake‐dwelling salmonids and that lakes are far less exposed to micropollutants than rivers and streams (e.g., Moschet et al., 2013). Lakes tend to display lower concentration of micropollutants than rivers because of an increased dilution factor, a longer residence time, and a higher degradation of the compounds (Moschet et al., 2013). Aerni et al. (2004) compared the estrogenic activity in sewage treatment plant effluents, rivers, and lakes of Switzerland and detected natural estrogens to be generally at a lower concentration in lakes than in rivers. We therefore predict that lake‐spawning and lake‐dwelling fish are more likely to show genetic variance for tolerance to this estrogen than river‐dwelling fish.

In order to investigate a potential erosion of additive genetic variance for tolerance to EE2, we sampled seven populations that show significant genetic and morphological differences (Stelkens, Jaffuel et al., 2012) and that inhabit rivers and streams of different ecology and different degrees of urbanization and effluent loads (i.e., rough proxies for levels of EE2 pollution). We found no interactions between treatment and population, that is, no population‐specific susceptibility to EE2. A possible explanation for this result is that all study populations had been exposed to more than a critical level of EE2 over periods that were always long enough to erode additive genetic variance for tolerance.

It would now be interesting to better understand whether and how ecologically relevant concentrations of EE2 (and other micropollutants) can erode genetic variance for tolerance in river‐dwelling salmonids. Such questions could potentially be addressed with experimental evolution on populations that (a) have never been exposed to EE2 (which is rare given the increasing human population density and the observation that very low concentrations of EE2 can induce selection) and (b) have never been mixed with populations that had been exposed to EE2 (which is challenging because salmonids are charismatic and economically relevant species, and stocking is widespread and hard to control by authorities). Alternatively, genomic variation could be examined for signatures of selection in the EE2 response pathways, analogously to studies done on other organisms and other pollutants (Csilléry, Rodríguez‐Verdugo, Rellstab, & Guillaume, 2018; Osterberg, Cammen, Schultz, Clark, & Di Giulio, 2018; Weigand & Leese, 2018; Weigand et al., 2018).

To conclude, EE2 is a common pollutant in the aquatic environment (Tiedeken et al., 2017). Using an experimental protocol that circumvents many of the typical problems of ecotoxicological studies (Wedekind, von Siebenthal, & Gingold, 2007), and by sampling several populations, we found a low and ecologically relevant concentration of EE2 to produce detrimental effects on brown trout early life‐history traits. Interestingly, despite displaying high genetic diversity (Stelkens, Jaffuel et al., 2012; Stelkens, Pompini et al., 2012), we did not find any of the study populations to display additive genetic variance for tolerance to this pollutant. One possible explanation for this result is that previous exposure to EE2 has eroded additive genetic variance for tolerance.

## CONFLICT OF INTEREST

None declared.

## AUTHOR CONTRIBUTIONS

LMC, AU, EV, and CW designed the experiments. LMC, AU, DN, and CW did the in vitro fertilizations and distributed the fertilized eggs to the 24‐well plates. LMC and AU treated and monitored the embryos. ES and DN determined larval growth. LMC sampled water for chemical analyses that were supervised by EV. LMC and CW analyzed the data and wrote the manuscript that was critically revised by all authors.

## Data Availability

The data used in this study have been deposited on the Dryad repository https://doi.org/10.5061/dryad.s7355g5.
