# Supplementary material for: No additive genetic variance for tolerance to ethynylestradiol exposure in natural populations of brown trout (Salmo trutta)
Source: Evol Appl. 2019 Jan 28;12(5):940–50. doi: 10.1111/eva.12767 (PMC6503824; doi:10.1111/eva.12767)
Supplement: Supplementary file 1 [file EVA-12-940-s001.docx]

**Supplementary information**

**No additive genetic variance for tolerance to ethynylestradiol** **exposure in natural populations of brown trout (*Salmo trutta*)**

*Quantification of estrone, 17***β***-estradiol and bisphenol A from 24 well-plates water*

Estrone (E1), 17β-estradiol (E2) and bisphenol A (BPA) were quantified in water from 24 well-plates as part of the method to determine 17**α**-ethynylestradiol (EE2) in experiment 2 of the main manuscript. For EE2, E1, E2, and BPA mass transitions, see Table S2.

Concentrations of E1 and E2 above the limit of quantification were detected at a very early stage, in the first week of development, and increased until 8 and 4 weeks post fertilization, respectively (Table S3a–b). Towards hatching, the concentration of both natural steroids sharply decreased (Table S3a–b), indicating embryo metabolism or uptake. However, the appearance of E1 and E2 does not seem to be linked to the EE2 treatment to which embryos were exposed. These natural steroids were not found above the limit of quantification in plates without embryos. Our results support E1 and E2 metabolism patterns described in rainbow trout (*Oncorhynchus mykiss*) embryos (Petkam et al., 2002). During experiment 2, which we performed to determine exposure conditions in experiment 1, we detected significant levels of BPA in plates with and without embryos. BPA is a synthetic chemical compound that leaches from several types of plastics and is a widely spread endocrine-disrupting chemical (Corrales et al., 2015). In fish, BPA is around 300 times less estrogenic than EE2 (reviewed in Segner et al., 2003). Moreover, the reported levels of BPA did not bias the interpretation of our results because we did not measure biological traits of embryos in experiment two. An additional (third) experiment performed after the reported BPA detections, and with the goal of tracking the BPA source, revealed that this contamination was temporary because no abnormal levels of BPA were measured (BPA concentrations always < 105 ng/L).

Table S1. Dates of sampling adults from the wild (all in 2014).

| **Population** | **Sampling dates** |
| --- | --- |
| Aare | October 27 and 28, November 3 |
| Giesse | November 1 |
| Gürbe | October 23 |
| Kiese | October 28 |
| Müsche | November 13 and 14 |
| Rotache | October 24 |
| Worble | November 4 |

Table S2. Details on the sample size across populations, individuals and treatments.

| **Population** | **N_breeding blocks_** | **N_females_** | **N_males_** | **N_families_** | **N_embryos_** | **N_embryos control_** | **N_embryos EE2_** |
| --- | --- | --- | --- | --- | --- | --- | --- |
| Aare | 1 | 4 | 5 | 20 | 200 | 100 | 100 |
| Giesse | 3 | 13 | 15 | 65 | 651 | 325 | 326 |
| Gürbe | 7 | 36 | 34 | 180 | 1800 | 900 | 900 |
| Kiese | 2 | 10 | 10 | 50 | 500 | 250 | 250 |
| Müsche | 6 | 32 | 31 | 165 | 1651 | 826 | 825 |
| Rotache | 5 | 25 | 24 | 125 | 1250 | 625 | 625 |
| Worble | 5 | 25 | 23 | 125 | 1250 | 625 | 625 |
| **Total** | **29** | **145** | **142** | **730** | **7302** | **3651** | **3651** |

Table S3. Mass transitions for quantifying, 17**α-**ethynylestradiol (EE2), bisphenol A (BPA), estrone (E1), and 17β-estradiol (E2), as well as their deuterated (D) internal standards.

| Compound | Retention time (min) | Transition quantifier | Transition qualifier |
| --- | --- | --- | --- |
| EE2 | 8.73 | 295 🡪 269 | 295 🡪 199 |
| EE2-D4 | 8.69 | 299 🡪 273 | 299 🡪 147 |
| BPA | 6.41 | 227 🡪 212 | 227 🡪 133 |
| BPA-D16 | 6.38 | 231 🡪 216 | 231 🡪 135 |
| E1 | 7.80 | 269 🡪 159 | 269 🡪 145 |
| E1-D4 | 7.77 | 273 🡪 161 | 273 🡪 147 |
| E2 | 8.55 | 271 🡪 239 | 271 🡪 143 |
| E2-D4 | 8.51 | 275 🡪 187 | 275 🡪 147 |

Table S4. Measured concentrations of (A) estrone (E1), (B) 17β-estradiol (E2) and (C) bisphenol A (BPA) in water samples from 24-well plates with or without brown trout embryos. Samples were collected at 5 time points during embryo development: the day of EE2 exposure (i.e. day 1 post fertilization) and then 7, 28, 56, and 84 days post fertilization.

| Days after exposure | No embryos  Control | No embryos EE2 | With embryos  Control | With embryos EE2 |
| --- | --- | --- | --- | --- |
| *(A) E1(ng/L)* |  |  |  |  |
| 1 | <0.05 | <0.05 | <0.05 | <0.05 |
| 7 | <0.05 | <0.05 | 1.6 | 2.3 |
| 28 | <0.05 | <0.05 | 13 | 12 |
| 56 | <0.05 | <0.05 | 12 | 11 |
| 84 | <0.05 | <0.05 | 4.2 | 3.8 |
|  |  |  |  |  |
| *(B) E2 (ng/L)* |  |  |  |  |
| 1 | <0.05 | <0.05 | <0.05 | <0.05 |
| 7 | <0.05 | <0.05 | 3.3 | 3.8 |
| 28 | <0.05 | <0.05 | 2.3 | 1.5 |
| 56 | <0.1 | <0.1 | 0.5 | 0.5 |
| 84 | <0.1 | <0.1 | 0.5 | 0.5 |
|  |  |  |  |  |
| *(C) BPA (ng/L)* |  |  |  |  |
| 1 | 8100 | 9500 | 8100 | 9500 |
| 7 | 7600 | 9900 | 8100 | 9000 |
| 28 | 9100 | 8800 | 4900 | 5800 |
| 56* | 7800 | 8500 | 2700 | 2700 |
| 84* | 8700 | 9200 | 900 | 900 |

* As samples contained high concentrations of BPA they did not require enrichment but could be injected directly (together with internal standard) into the LC-MS/MS.

FIGURE S1: Experimental breeding, sampling, and raising of embryos and larvae. (A) Eggs (yellow to orange in the photo) and milt (white drops next to the eggs) were used for full-factorial *in vitro* breeding experiments that mostly involved the eggs of 5 females distributed to 5 Petri dishes (ordered in rows here) and the drops of milt of mostly five males that were used to fertilize one batch of eggs per females, so that all possible sib groups were produced. (B) After sperm had been activated by adding water, the fertilized eggs were given 2 hours to harden before a sample of fertilized eggs of each sib group was taken into the laboratory and distributed to 24-well polystyrene plates. About half of these freshly fertilized eggs per sib group were then exposed to EE2, the other ones were sham exposed. (C) After hatching, the yolk-sac larvae were transferred to 12-well plates to simplify growth measurements.


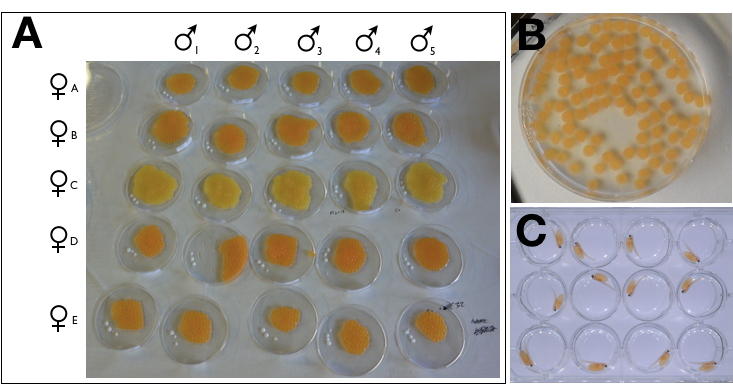


FIGURE S2: Example of the larval measurements taken for each individual in the day of hatching and 24 days later.


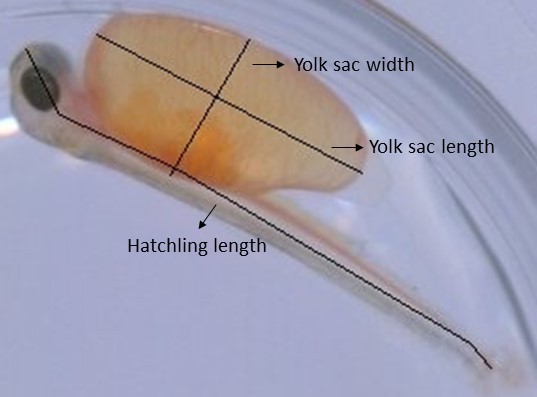


**References**

Corrales, J., Kristofco, L. A., Steele, W. B., Yates, B. S., Breed, C. S., Williams, E. S. & Brooks, B. W. 2015. Global assessment of bisphenol A in the environment: review and analysis of its occurrence and bioaccumulation. *Dose-Response* **13**: 1559325815598308.

Petkam, R., Renaud, R., Freitas, A., Canario, A. V. & Leatherland, J. 2002. In vitro metabolism of progesterone, androgens and estrogens by rainbow trout embryos. *Fish Physiology and Biochemistry* **27**: 117-128.

Segner, H., Navas, J., Schäfers, C. & Wenzel, A. 2003. Potencies of estrogenic compounds in in vitro screening assays and in life cycle tests with zebrafish in vivo. *Ecotoxicology and Environmental Safety* **54**: 315-322.
